# Acute abdomen in a 54-year-old COVID-19 patient: a case teport

**DOI:** 10.1093/jscr/rjab198

**Published:** 2021-05-28

**Authors:** Peter Holleb, Priya Patel, Pranay Saxena, Jagbir Beniwal, Jamshed Zuberi

**Affiliations:** St. George’s University School of Medicine, True Blue, Grenada, West Indies; St. George’s University School of Medicine, True Blue, Grenada, West Indies; Department of Surgery, CarePoint Health, Bayonne, NJ, USA; Department of Surgery, St. Joseph’s University Medical Center, Paterson, NJ, USA; Department of Surgery, St. Joseph’s University Medical Center, Paterson, NJ, USA

## Abstract

Although primarily a respiratory virus, coronavirus-19 acts on the gastrointestinal tract to cause symptoms such as anorexia, nausea, vomiting and diarrhea. One possible mechanism involves the ACE2 receptor, which serves as the primary receptor for virus entry into the gastrointestinal epithelium. We describe the case of a 54-year-old-male with recent coronavirus disease 2019 (COVID-19) infection, who later presented with nausea, vomiting, diarrhea and progressively worsening diffuse abdominal pain for 1 week. He was diagnosed to have a small bowel obstruction; however, continued to have progressively worsening pain and failed conservative management. No cause for the obstruction was found in the operating room. Gastrointestinal involvement occurs in at least two-thirds of patients with coronavirus infection. Viral entry into the small bowel, triggering an inflammatory response, and virus-induced microthrombosis of the microcirculation have been postulated as a possible mechanism for paralytic ileus/small bowel obstruction.

## BACKGROUND

Coronavirus disease 2019 (COVID-19) is caused by severe acute respiratory syndrome coronavirus 2 (SARS-CoV-2). Although primarily a respiratory virus, it acts on the gastrointestinal (GI) tract to cause symptoms such as anorexia, nausea, vomiting and diarrhea [1]. One possible mechanism for gastrointestinal symptoms involves the angiotensin converting enzyme 2 (ACE2) receptor. The ACE2 protein serves as the primary receptor for virus entry into the gastric, duodenal and rectal epithelium. These receptors are most prominent in the small and large intestine [2]. It has been hypothesized that the ACE2 receptors allow initial entry of COVID-19 to mediate inflammation of the large and small bowel [1]. More serious complications with critically ill patients include bowel ischemia, transaminitis, gastrointestinal bleeding, pancreatitis and ogilvie syndrome [3].

Small bowel obstruction (SBO) refers to a partial or complete blockage that prevents progression of abdominal contents through the intestinal tract. The most frequent causes include postoperative adhesions, hernias and neoplasms. Less common causes include intussusception, volvulus, Crohn’s disease and appendicitis. The predominant symptoms are obstipation/constipation, abdominal distension and vomiting (often bilious). Diagnosis is made through physical examination and imaging. On computerized tomography (CT) scan, SBO is identified with dilated loops of bowel (>2.5 cm) proximally and collapsed loops distally [4]. Findings on imaging that indicate complicated SBO include abdominal free air, bowel wall thickening, and reduced enhancement [4]. Complications include bowel ischemia, necrosis and perforation. Initial treatment consists of fluid therapy, correction of electrolyte imbalances and nasogastric decompression. Severe complications including peritonitis or free air or lack or improvement with conservative management require surgical intervention.

We describe the case of a patient who presented with a small bowel obstruction possibly due to a COVID-19-related ileus. In the operating room, a cause for the obstruction could not be found.

## CASE DESCRIPTION

**Figure 1:**
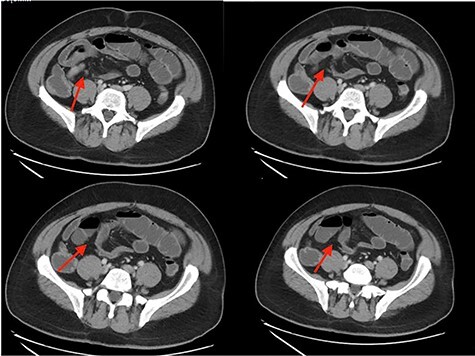
CT abdomen/pelvis showing SBO with transition point in right lower quadrant.

This case is a 54-year-old-male with past medical history of obesity, laparoscopic cholecystectomy, recurrent small bowel obstructions in 1997 and 2006 resolved with supportive care and COVID-19 infection in December 2020 who presented to the Emergency Department with nausea, non-bilious vomiting, non-bloody diarrhea and progressively worsening, sharp, diffuse abdominal pain for 1 week. On physical examination, he had diffuse abdominal tenderness. His white blood cell count was 3.8, consistent with his recent COVID-19 infection. Abdominal CT showed partial small bowel obstruction with a transition point in the right lower quadrant as well as fluid in the colon and throughout the bowel (Fig. 1). The patient had two episodes of bilious emesis overnight after his admission. A nasogastric tube was inserted and conservative management was attempted. The patient had persistently worsening abdominal pain, and was taken to the operating room for an exploratory laparotomy 2 days after admission.

In the operating room, a midline longitudinal incision was made. The small bowel was found to be dilated but there was no definite transition point of obstruction found. There were some adhesions in the right lower quadrant around the appendix and the cecum, but no obstruction was found.

**Figure 2:**
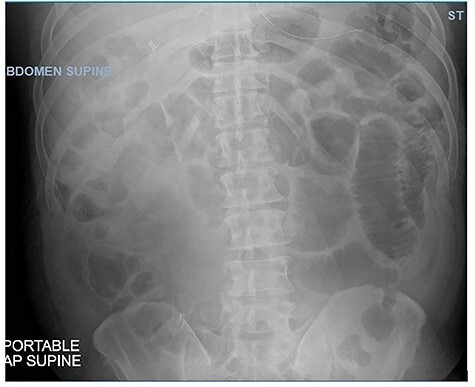
Abdominal X-ray showing dilated loops of bowel on Day 9 of admission.

Postoperatively, the patient remained NPO with an NG tube for 8 days. On Day 9, his abdominal X-ray (Fig. 2) was much improved and without air-fluid levels, he was passing flatus, and his abdominal exam was clinically benign. He was started on a clear liquid diet and advanced to a regular diet over the next day. The patient was discharged 13 days after the admission. He was tolerating his diet and had regular bowel function with no further symptoms of abdominal pain, nausea or vomiting.

## DISCUSSION

Gastrointestinal involvement occurs in at least two-thirds of patients with SARS-CoV-2 infection [1]. It enters the small bowel by interaction with ACE2 receptors and triggers an inflammatory response (cytokine storm) and disrupts the gut microbiome [3]. The epithelial cells within the lymphoid tissue of the small bowel act as sensors in response to viral entry and secrete TNF and IL-6 [5]. This results in the recruitment of neutrophils, monocytes, phagocytic macrophages and T cells in the cytokine storm known to occur in SARS and COVID-19 [6]. This inflammation has been postulated as a possible mechanism for paralytic ileus.

Another possible mechanism of paralytic ileus includes virus-induced microthrombosis of the microcirculation resulting in dilation within the large and small bowel [7]. Viral infection of the endothelial cells creates diffuse endothelial inflammation and increases procoagulant factors like factor VII, fibrinogen and von Willebrand factor in addition to the virus-induced cytokine storm leading to fibrinolysis [8]. In addition, a theory for the propagation of hypercoagulability involves the neutrophil extracellular traps (NETs) [8]. Normally, they function to control infections; however, when not properly modulated, they exacerbate inflammation and microvascular thrombosis, especially in the GI tract [8].

Previously published cases include an 80-year-old female who presented with a 10-day course of fevers, chills and productive cough [9]. On physical examination, she had decreased breath sounds on the right lung with diffuse rhonchi, abdominal pain, guarding and rebound [9]. After stabilization IV fluids, she underwent exploratory laparotomy due to evidence of reduced visceral perfusion in gastrointestinal tract and was found to have four punctate lesions in the sigmoid colon [9]. She was started on broad-spectrum antibiotics on admission; however, she went into septic shock and died on postoperative Day 2. Pathology showed ulcerated and perforated colonic segmental necrosis [9].

Another case involved a 53-year-old male patient who presented with abdominal discomfort and distension, progressively worsening obstipation and fever that had worsened over 2 days [10]. Abdominal radiographs showed distention of the colonic loops [10]. He underwent an exploratory laparotomy. Lab studies showed lymphopenia and his COVID-19 test was positive. These findings are consistent with our patient who also showed a lower white blood cell (WBC) count with dilated bowel loops.

Possible causes of COVID-19-related ileus include hypercoagulability causing microthromboses and inflammation mediated through ACE2 receptors. Patients present with abdominal pain and distention, nausea, vomiting, obstipation, guarding, rebound and/or peritonitis. Alter the management of patients with gastrointestinal predominant symptoms, starting them on a bowel regimen and anticoagulation early could prevent small bowel obstruction or ileus and prevent its complications.
