# Erratum to: Acute abdomen in a 54-year-old COVID-19 patient: a case report

**DOI:** 10.1093/jscr/rjac089

**Published:** 2022-03-08

**Authors:** Peter Holleb, Priya Patel, Pranay Saxena, Jagbir Beniwal, Jamshed Zuberi

**Affiliations:** 1 St. George’s University School of Medicine, True Blue, Grenada, West Indies; 2 Department of Surgery, CarePoint Health, Bayonne, NJ, USA; 3 Department of Surgery, St. Joseph’s University Medical Center, Paterson, NJ, USA

In the originally published version of this manuscript, there was an error in the title. The title should read: ``Acute abdomen in a 54-year-old COVID-19 patient: a case report'' instead of ``Acute abdomen in a 54-year-old COVID-19 patient: a case teport''. This error has been corrected online.

